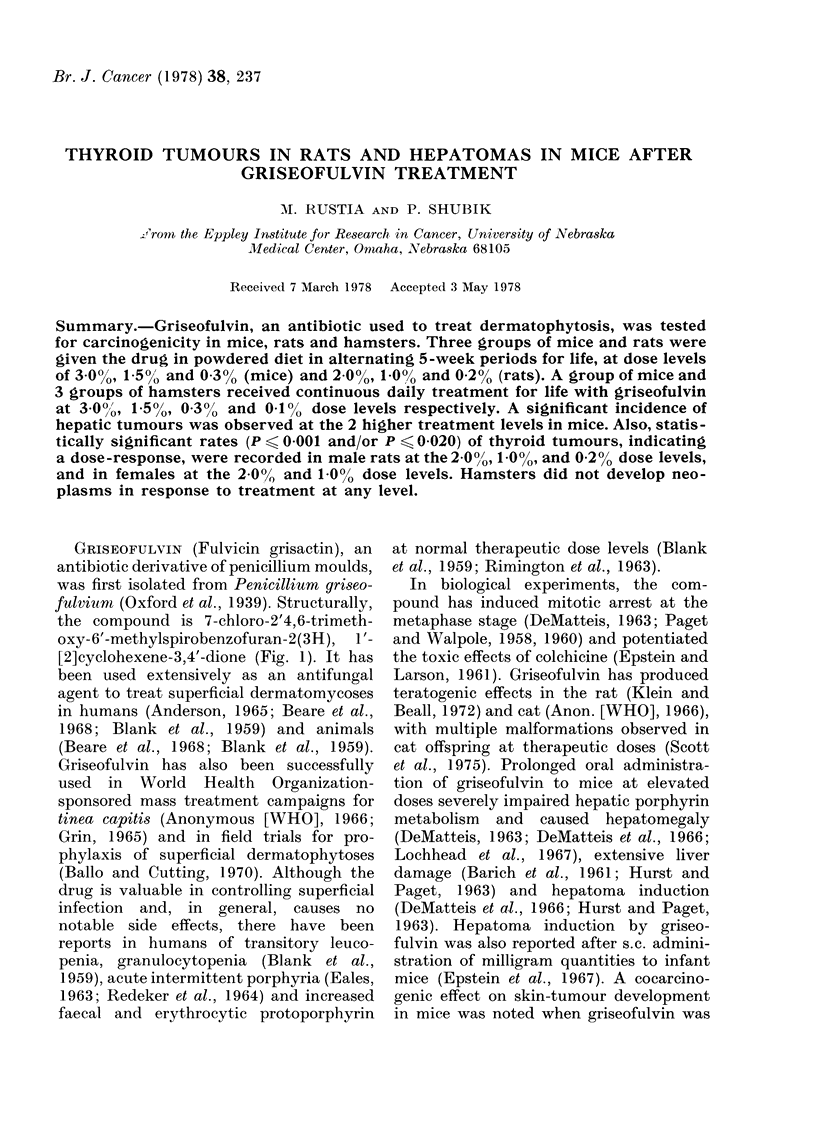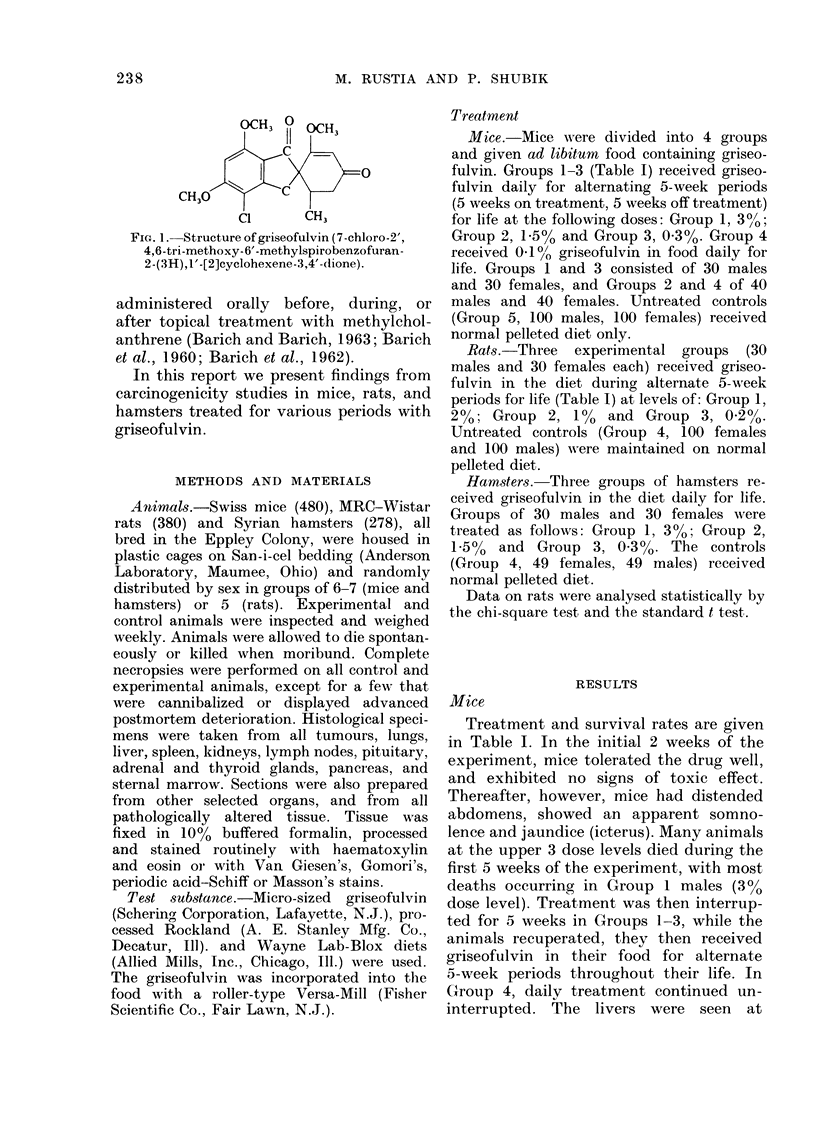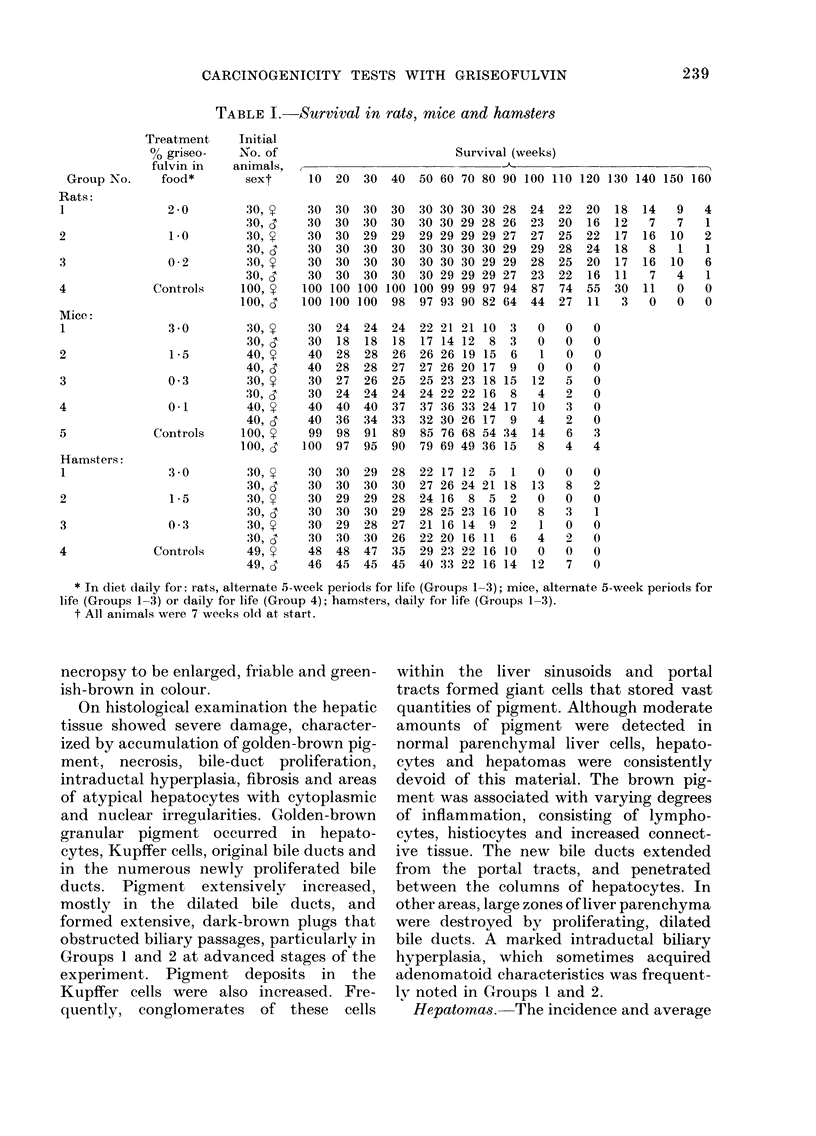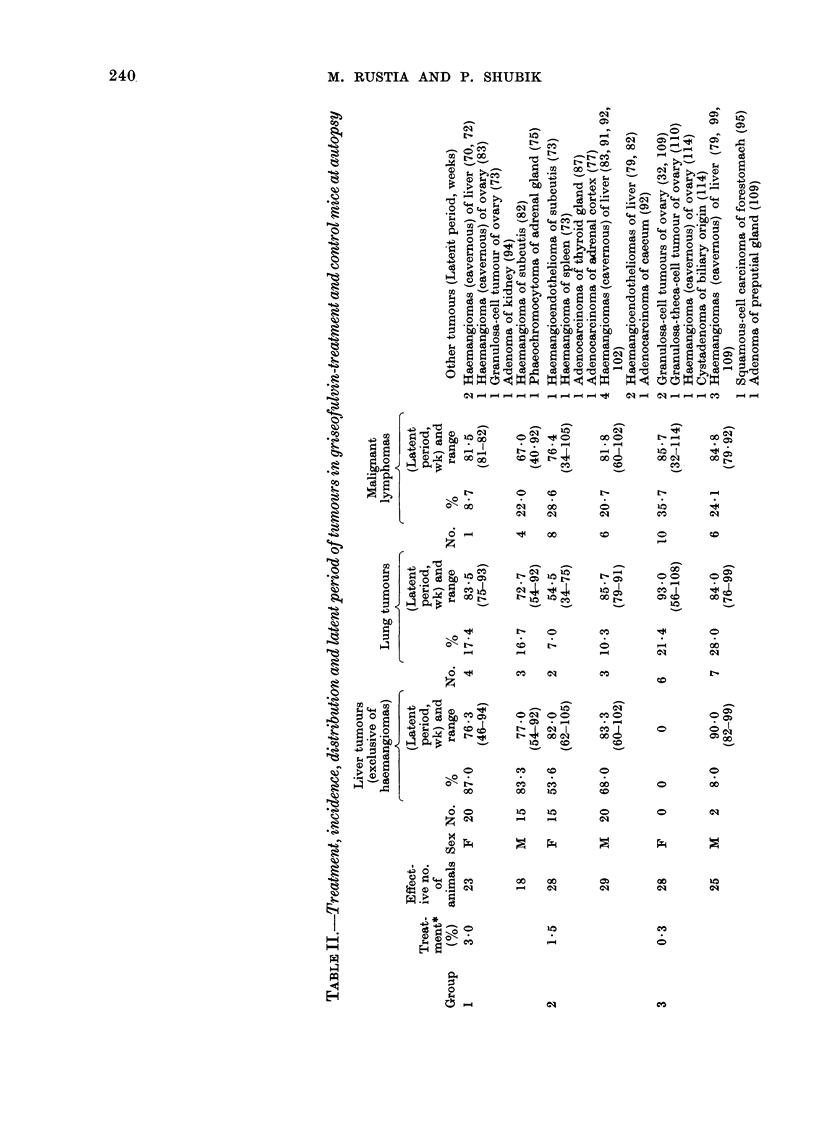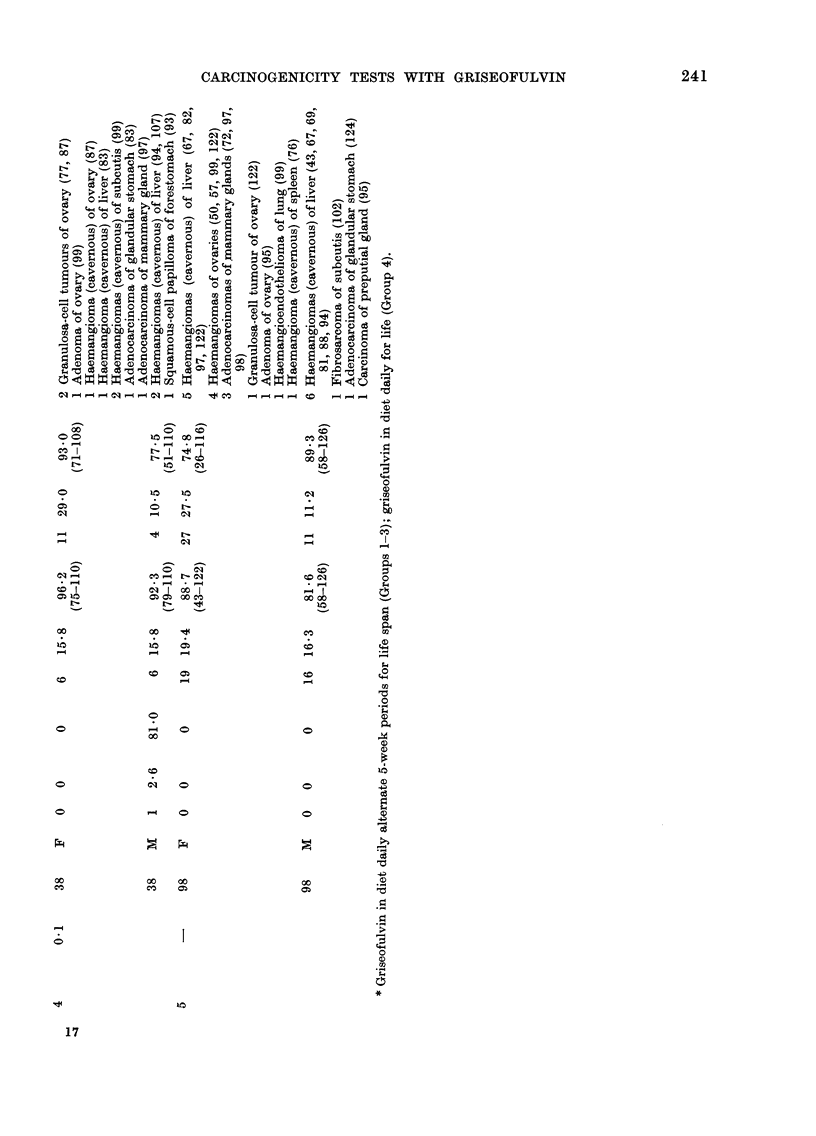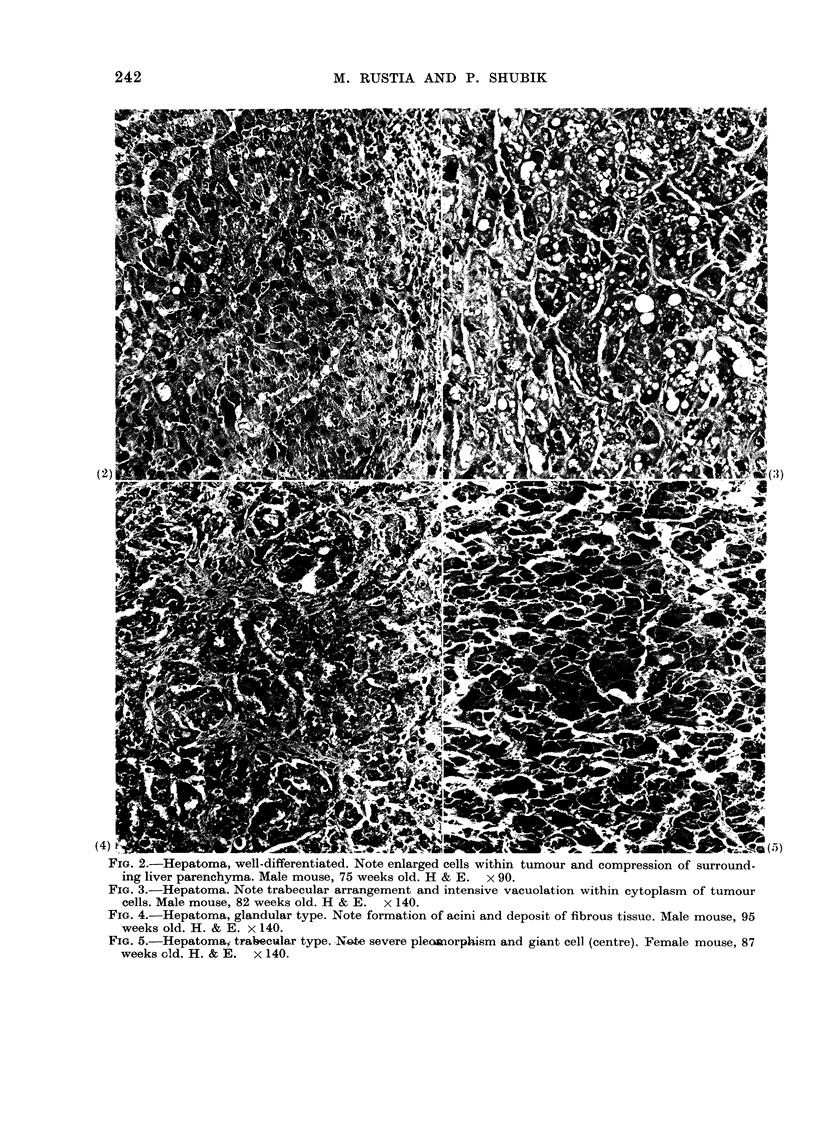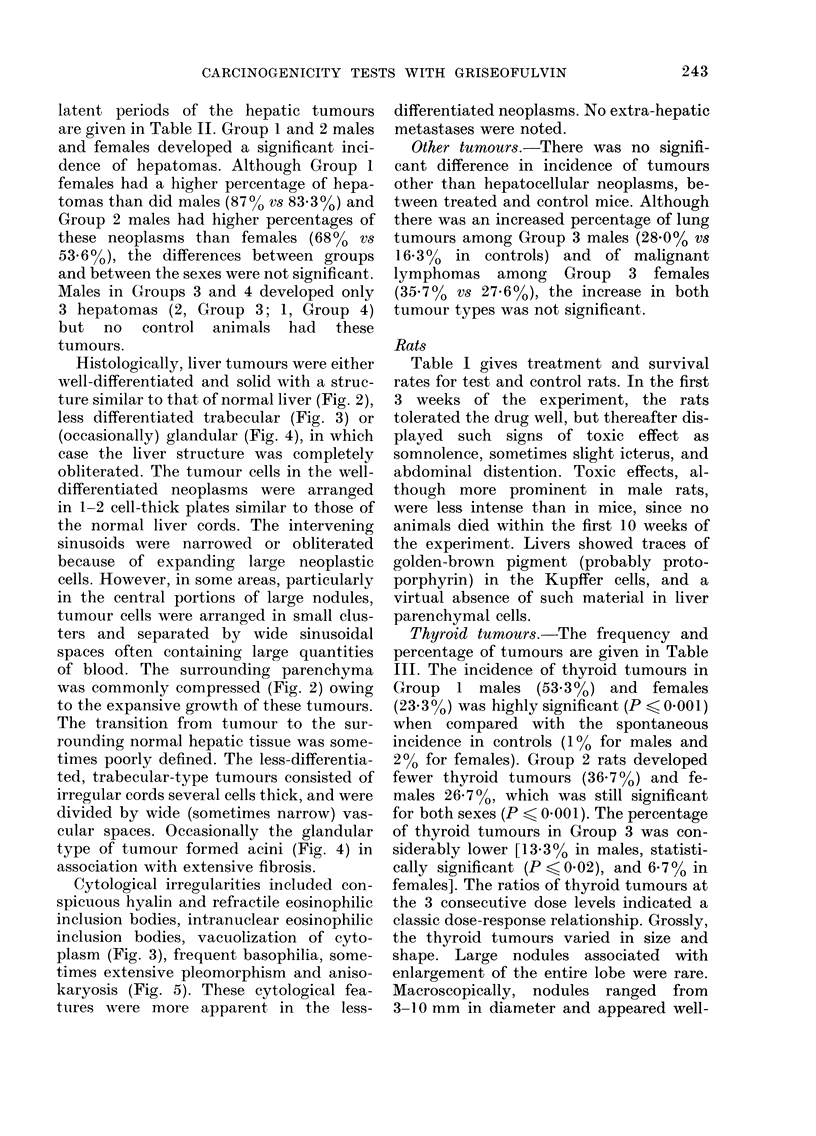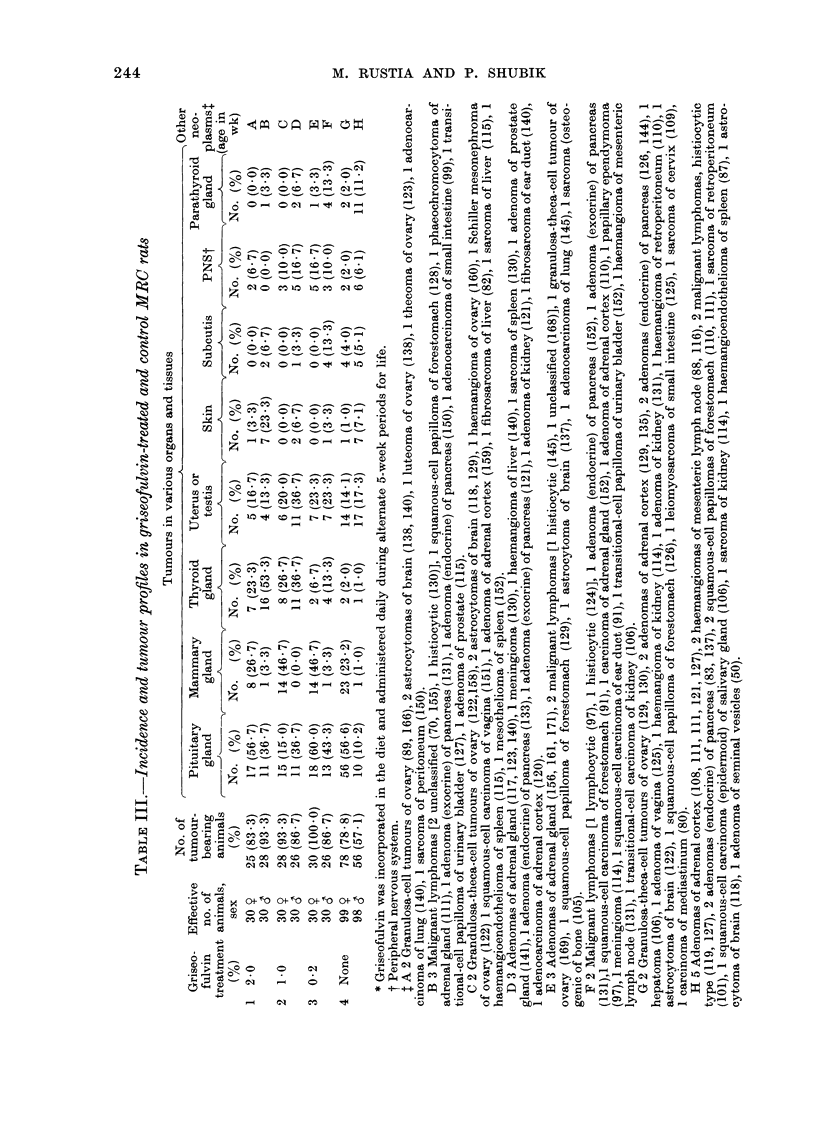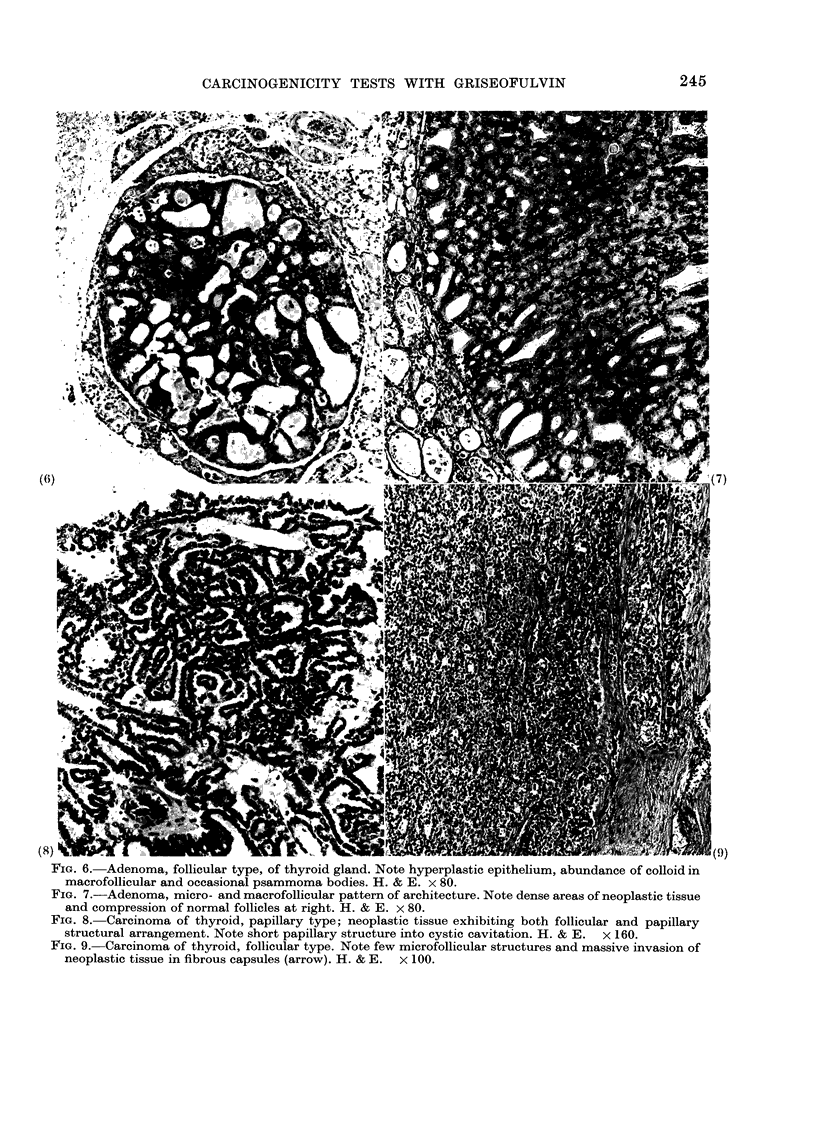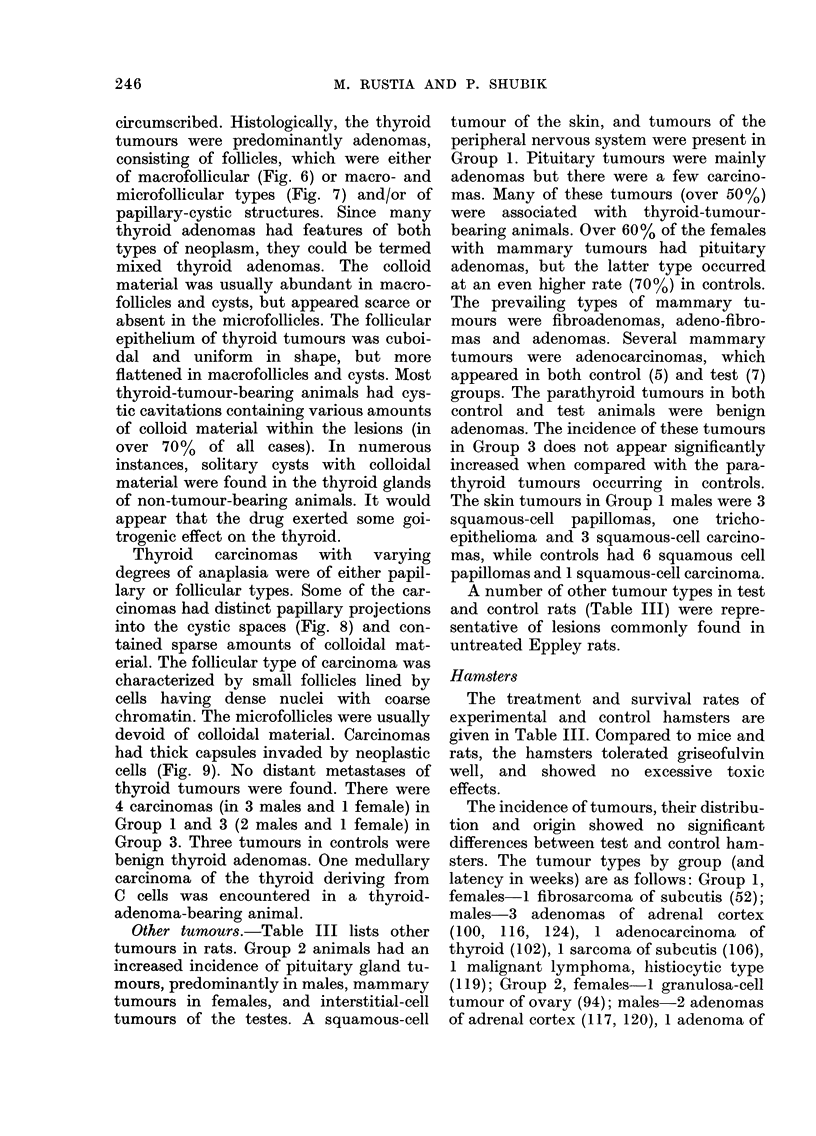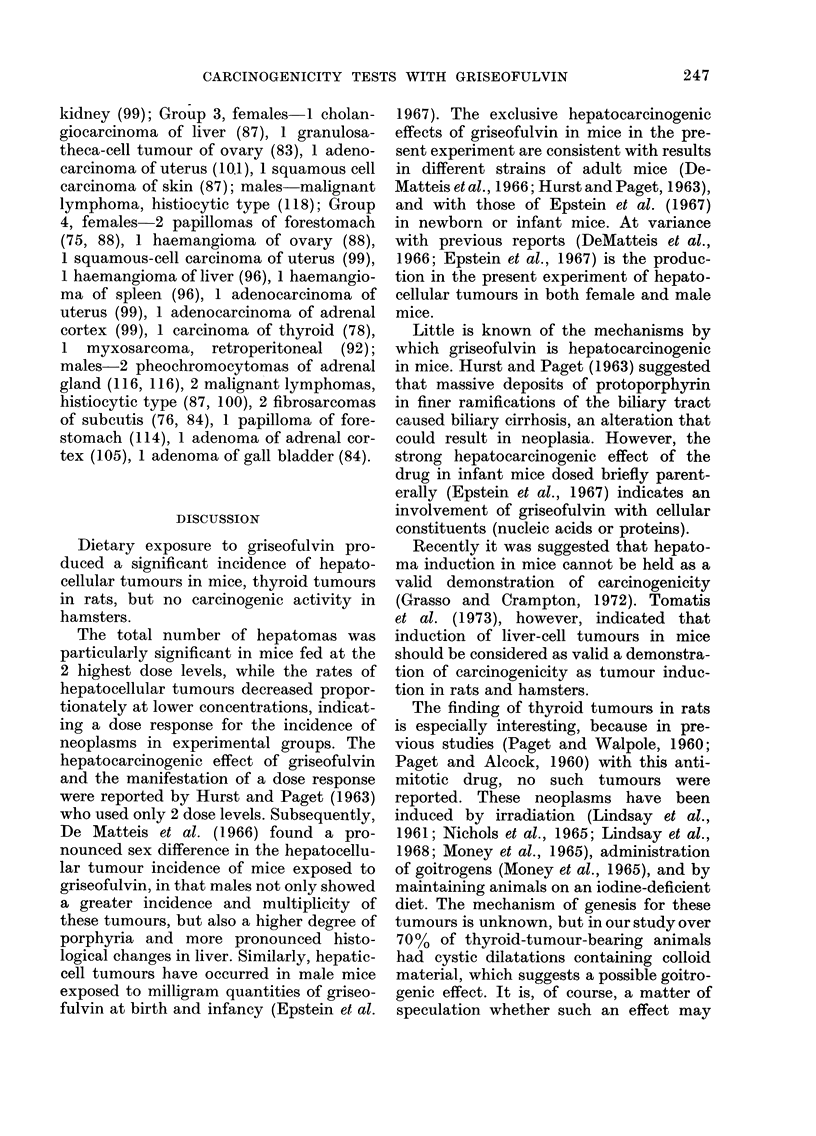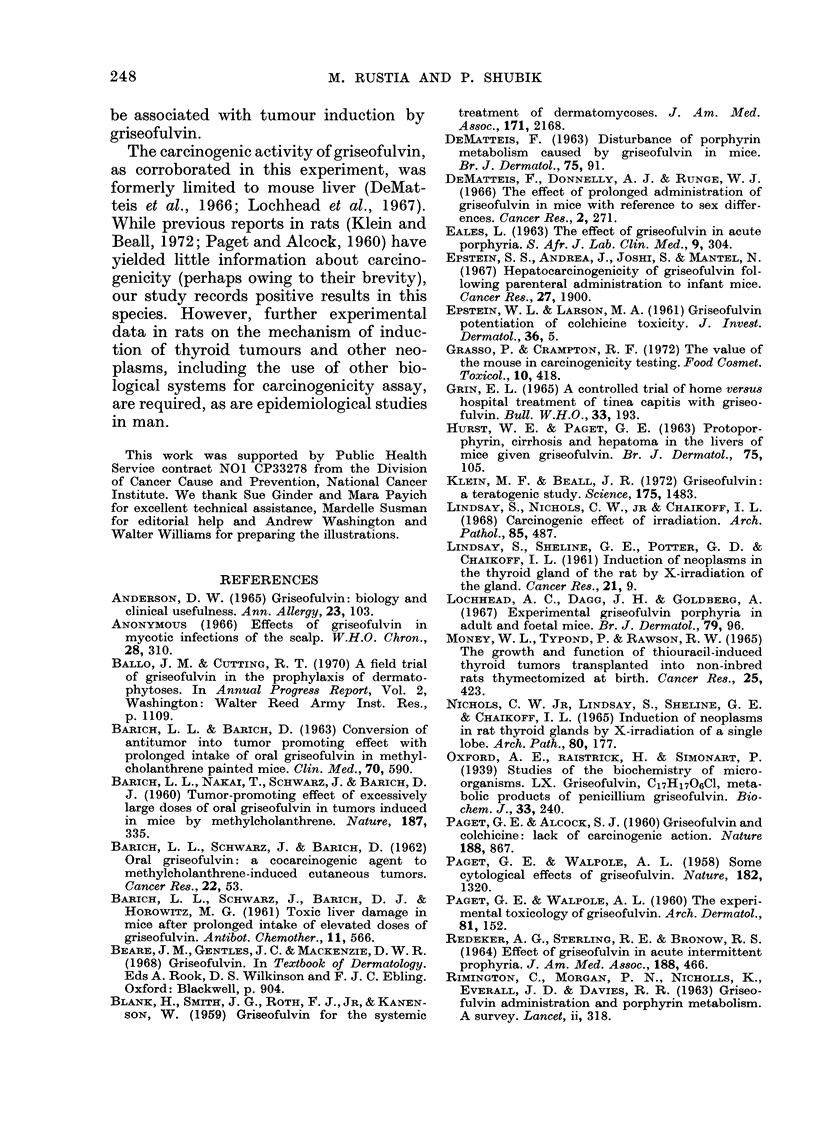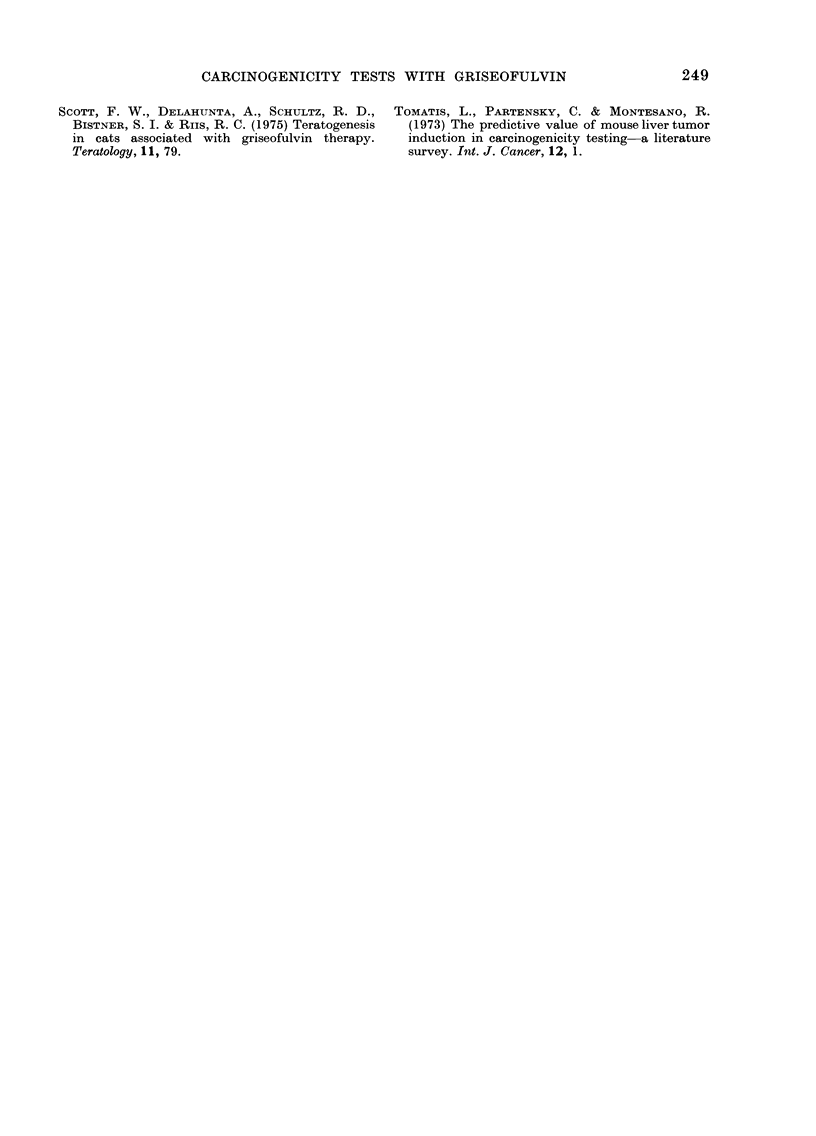# Thyroid tumours in rats and hepatomas in mice after griseofulvin treatment.

**DOI:** 10.1038/bjc.1978.194

**Published:** 1978-08

**Authors:** M. Rustia, P. Shubik

## Abstract

**Images:**


*Br. J. Cancer* (1978) **38**, 237

### THYROID TUMOURS IN RATS AND HEPATOMAS IN MICE AFTER GRISEOFULVIN TREATMENT

M. RUSTIA AND P. SHUBIK

*From the Eppley Institute for Research in Cancer, University of Nebraska Medical Center, Omaha, Nebraska* 68105

Received 7 March 1978 Accepted 3 May 1978

**Summary.—Griseofulvin, an antibiotic used to treat dermatophytosis, was tested for carcinogenicity in mice, rats and hamsters. Three groups of mice and rats were given the drug in powdered diet in alternating 5-week periods for life, at dose levels of 3·0%, 1·5% and 0·3% (mice) and 2·0%, 1·0% and 0·2% (rats). A group of mice and 3 groups of hamsters received continuous daily treatment for life with griseofulvin at 3·0%, 1·5%, 0·3% and 0·1% dose levels respectively. A significant incidence of hepatic tumours was observed at the 2 higher treatment levels in mice. Also, statistically significant rates ($P \leqslant 0.001$ and/or $P \leqslant 0.020$) of thyroid tumours, indicating a dose-response, were recorded in male rats at the 2·0%, 1·0%, and 0·2% dose levels, and in females at the 2·0% and 1·0% dose levels. Hamsters did not develop neoplasms in response to treatment at any level.**

GRISEOFULVIN (Fulvicin grisactin), an antibiotic derivative of penicillium moulds, was first isolated from *Penicillium griseofulvium* (Oxford *et al.*, 1939). Structurally, the compound is 7-chloro-2′4,6-trimethoxy-6′-methylspirobenzofuran-2(3H), 1′-[2]cyclohexene-3,4′-dione (Fig. 1). It has been used extensively as an antifungal agent to treat superficial dermatomycoses in humans (Anderson, 1965; Beare *et al.*, 1968; Blank *et al.*, 1959) and animals (Beare *et al.*, 1968; Blank *et al.*, 1959). Griseofulvin has also been successfully used in World Health Organization-sponsored mass treatment campaigns for *tinea capitis* (Anonymous [WHO], 1966; Grin, 1965) and in field trials for prophylaxis of superficial dermatophytoses (Ballo and Cutting, 1970). Although the drug is valuable in controlling superficial infection and, in general, causes no notable side effects, there have been reports in humans of transitory leucopenia, granulocytopenia (Blank *et al.*, 1959), acute intermittent porphyria (Eales, 1963; Redeker *et al.*, 1964) and increased faecal and erythrocytic protoporphyrin at normal therapeutic dose levels (Blank *et al.*, 1959; Rimington *et al.*, 1963).

In biological experiments, the compound has induced mitotic arrest at the metaphase stage (DeMatteis, 1963; Paget and Walpole, 1958, 1960) and potentiated the toxic effects of colchicine (Epstein and Larson, 1961). Griseofulvin has produced teratogenic effects in the rat (Klein and Beall, 1972) and cat (Anon. [WHO], 1966), with multiple malformations observed in cat offspring at therapeutic doses (Scott *et al.*, 1975). Prolonged oral administration of griseofulvin to mice at elevated doses severely impaired hepatic porphyrin metabolism and caused hepatomegaly (DeMatteis, 1963; DeMatteis *et al.*, 1966; Lochhead *et al.*, 1967), extensive liver damage (Barich *et al.*, 1961; Hurst and Paget, 1963) and hepatoma induction (DeMatteis *et al.*, 1966; Hurst and Paget, 1963). Hepatoma induction by griseofulvin was also reported after s.c. administration of milligram quantities to infant mice (Epstein *et al.*, 1967). A cocarcinogenic effect on skin-tumour development in mice was noted when griseofulvin was

FIG. 1.—Structure of griseofulvin (7-chloro-2′, 4,6-tri-methoxy-6′-methylspirobenzofuran-2-(3H),1′-[2]cyclohexene-3,4′-dione).

administered orally before, during, or after topical treatment with methylcholanthrene (Barich and Barich, 1963; Barich *et al.*, 1960; Barich *et al.*, 1962).

In this report we present findings from carcinogenicity studies in mice, rats, and hamsters treated for various periods with griseofulvin.

### METHODS AND MATERIALS

*Animals.*—Swiss mice (480), MRC–Wistar rats (380) and Syrian hamsters (278), all bred in the Eppley Colony, were housed in plastic cages on San-i-cel bedding (Anderson Laboratory, Maumee, Ohio) and randomly distributed by sex in groups of 6–7 (mice and hamsters) or 5 (rats). Experimental and control animals were inspected and weighed weekly. Animals were allowed to die spontaneously or killed when moribund. Complete necropsies were performed on all control and experimental animals, except for a few that were cannibalized or displayed advanced postmortem deterioration. Histological specimens were taken from all tumours, lungs, liver, spleen, kidneys, lymph nodes, pituitary, adrenal and thyroid glands, pancreas, and sternal marrow. Sections were also prepared from other selected organs, and from all pathologically altered tissue. Tissue was fixed in 10% buffered formalin, processed and stained routinely with haematoxylin and eosin or with Van Giesen's, Gomori's, periodic acid–Schiff or Masson's stains.

*Test substance.*—Micro-sized griseofulvin (Schering Corporation, Lafayette, N.J.), processed Rockland (A. E. Stanley Mfg. Co., Decatur, Ill). and Wayne Lab-Blox diets (Allied Mills, Inc., Chicago, Ill.) were used. The griseofulvin was incorporated into the food with a roller-type Versa-Mill (Fisher Scientific Co., Fair Lawn, N.J.).

### *Treatment*

*Mice.*—Mice were divided into 4 groups and given *ad libitum* food containing griseofulvin. Groups 1–3 (Table I) received griseofulvin daily for alternating 5-week periods (5 weeks on treatment, 5 weeks off treatment) for life at the following doses: Group 1, 3%; Group 2, 1·5% and Group 3, 0·3%. Group 4 received 0·1% griseofulvin in food daily for life. Groups 1 and 3 consisted of 30 males and 30 females, and Groups 2 and 4 of 40 males and 40 females. Untreated controls (Group 5, 100 males, 100 females) received normal pelleted diet only.

*Rats.*—Three experimental groups (30 males and 30 females each) received griseofulvin in the diet during alternate 5-week periods for life (Table I) at levels of: Group 1, 2%; Group 2, 1% and Group 3, 0·2%. Untreated controls (Group 4, 100 females and 100 males) were maintained on normal pelleted diet.

*Hamsters.*—Three groups of hamsters received griseofulvin in the diet daily for life. Groups of 30 males and 30 females were treated as follows: Group 1, 3%; Group 2, 1·5% and Group 3, 0·3%. The controls (Group 4, 49 females, 49 males) received normal pelleted diet.

Data on rats were analysed statistically by the chi-square test and the standard *t* test.

### RESULTS

### *Mice*

Treatment and survival rates are given in Table I. In the initial 2 weeks of the experiment, mice tolerated the drug well, and exhibited no signs of toxic effect. Thereafter, however, mice had distended abdomens, showed an apparent somnolence and jaundice (icterus). Many animals at the upper 3 dose levels died during the first 5 weeks of the experiment, with most deaths occurring in Group 1 males (3% dose level). Treatment was then interrupted for 5 weeks in Groups 1–3, while the animals recuperated, they then received griseofulvin in their food for alternate 5-week periods throughout their life. In Group 4, daily treatment continued uninterrupted. The livers were seen at

TABLE I.—*Survival in rats, mice and hamsters*

| Group No. | Treatment % griseofulvin in food* | Initial No. of animals, sex† | Survival (weeks) | | | | | | | | | | | | | | | |
|---|---|---|---|---|---|---|---|---|---|---|---|---|---|---|---|---|---|---|
| | | | 10 | 20 | 30 | 40 | 50 | 60 | 70 | 80 | 90 | 100 | 110 | 120 | 130 | 140 | 150 | 160 |
| Rats: | | | | | | | | | | | | | | | | | | |
| 1 | 2·0 | 30, ♀ | 30 | 30 | 30 | 30 | 30 | 30 | 30 | 30 | 28 | 24 | 22 | 20 | 18 | 14 | 9 | 4 |
| | | 30, ♂ | 30 | 30 | 30 | 30 | 30 | 30 | 29 | 28 | 26 | 23 | 20 | 16 | 12 | 7 | 7 | 1 |
| 2 | 1·0 | 30, ♀ | 30 | 30 | 29 | 29 | 29 | 29 | 29 | 29 | 27 | 27 | 25 | 22 | 17 | 16 | 10 | 2 |
| | | 30, ♂ | 30 | 30 | 30 | 30 | 30 | 30 | 30 | 30 | 29 | 29 | 28 | 24 | 18 | 8 | 1 | 1 |
| 3 | 0·2 | 30, ♀ | 30 | 30 | 30 | 30 | 30 | 30 | 30 | 29 | 29 | 28 | 25 | 20 | 17 | 16 | 10 | 6 |
| | | 30, ♂ | 30 | 30 | 30 | 30 | 30 | 29 | 29 | 29 | 27 | 23 | 22 | 16 | 11 | 7 | 4 | 1 |
| 4 | Controls | 100, ♀ | 100 | 100 | 100 | 100 | 100 | 99 | 99 | 97 | 94 | 87 | 74 | 55 | 30 | 11 | 0 | 0 |
| | | 100, ♂ | 100 | 100 | 100 | 98 | 97 | 93 | 90 | 82 | 64 | 44 | 27 | 11 | 3 | 0 | 0 | 0 |
| Mice: | | | | | | | | | | | | | | | | | | |
| 1 | 3·0 | 30, ♀ | 30 | 24 | 24 | 24 | 22 | 21 | 21 | 10 | 3 | 0 | 0 | 0 | | | | |
| | | 30, ♂ | 30 | 18 | 18 | 18 | 17 | 14 | 12 | 8 | 3 | 0 | 0 | 0 | | | | |
| 2 | 1·5 | 40, ♀ | 40 | 28 | 28 | 26 | 26 | 26 | 19 | 15 | 6 | 1 | 0 | 0 | | | | |
| | | 40, ♂ | 40 | 28 | 28 | 27 | 27 | 26 | 20 | 17 | 9 | 0 | 0 | 0 | | | | |
| 3 | 0·3 | 30, ♀ | 30 | 27 | 26 | 25 | 25 | 23 | 23 | 18 | 15 | 12 | 5 | 0 | | | | |
| | | 30, ♂ | 30 | 24 | 24 | 24 | 24 | 22 | 22 | 16 | 8 | 4 | 2 | 0 | | | | |
| 4 | 0·1 | 40, ♀ | 40 | 40 | 40 | 37 | 37 | 36 | 33 | 24 | 17 | 10 | 3 | 0 | | | | |
| | | 40, ♂ | 40 | 36 | 34 | 33 | 32 | 30 | 26 | 17 | 9 | 4 | 2 | 0 | | | | |
| 5 | Controls | 100, ♀ | 99 | 98 | 91 | 89 | 85 | 76 | 68 | 54 | 34 | 14 | 6 | 3 | | | | |
| | | 100, ♂ | 100 | 97 | 95 | 90 | 79 | 69 | 49 | 36 | 15 | 8 | 4 | 4 | | | | |
| Hamsters: | | | | | | | | | | | | | | | | | | |
| 1 | 3·0 | 30, ♀ | 30 | 30 | 29 | 28 | 22 | 17 | 12 | 5 | 1 | 0 | 0 | 0 | | | | |
| | | 30, ♂ | 30 | 30 | 30 | 30 | 27 | 26 | 24 | 21 | 18 | 13 | 8 | 2 | | | | |
| 2 | 1·5 | 30, ♀ | 30 | 29 | 29 | 28 | 24 | 16 | 8 | 5 | 2 | 0 | 0 | 0 | | | | |
| | | 30, ♂ | 30 | 30 | 30 | 29 | 28 | 25 | 23 | 16 | 10 | 8 | 3 | 1 | | | | |
| 3 | 0·3 | 30, ♀ | 30 | 29 | 28 | 27 | 21 | 16 | 14 | 9 | 2 | 1 | 0 | 0 | | | | |
| | | 30, ♂ | 30 | 30 | 30 | 26 | 22 | 20 | 16 | 11 | 6 | 4 | 2 | 0 | | | | |
| 4 | Controls | 49, ♀ | 48 | 48 | 47 | 35 | 29 | 23 | 22 | 16 | 10 | 0 | 0 | 0 | | | | |
| | | 49, ♂ | 46 | 45 | 45 | 45 | 40 | 33 | 22 | 16 | 14 | 12 | 7 | 0 | | | | |

* In diet daily for: rats, alternate 5-week periods for life (Groups 1–3); mice, alternate 5-week periods for life (Groups 1–3) or daily for life (Group 4); hamsters, daily for life (Groups 1–3).
† All animals were 7 weeks old at start.

necropsy to be enlarged, friable and greenish-brown in colour.

On histological examination the hepatic tissue showed severe damage, characterized by accumulation of golden-brown pigment, necrosis, bile-duct proliferation, intraductal hyperplasia, fibrosis and areas of atypical hepatocytes with cytoplasmic and nuclear irregularities. Golden-brown granular pigment occurred in hepatocytes, Kupffer cells, original bile ducts and in the numerous newly proliferated bile ducts. Pigment extensively increased, mostly in the dilated bile ducts, and formed extensive, dark-brown plugs that obstructed biliary passages, particularly in Groups 1 and 2 at advanced stages of the experiment. Pigment deposits in the Kupffer cells were also increased. Frequently, conglomerates of these cells within the liver sinusoids and portal tracts formed giant cells that stored vast quantities of pigment. Although moderate amounts of pigment were detected in normal parenchymal liver cells, hepatocytes and hepatomas were consistently devoid of this material. The brown pigment was associated with varying degrees of inflammation, consisting of lymphocytes, histiocytes and increased connective tissue. The new bile ducts extended from the portal tracts, and penetrated between the columns of hepatocytes. In other areas, large zones of liver parenchyma were destroyed by proliferating, dilated bile ducts. A marked intraductal biliary hyperplasia, which sometimes acquired adenomatoid characteristics was frequently noted in Groups 1 and 2.

*Hepatomas.*—The incidence and average

FIG. 2.—Hepatoma, well-differentiated. Note enlarged cells within tumour and compression of surrounding liver parenchyma. Male mouse, 75 weeks old. H & E. ×90.

FIG. 3.—Hepatoma. Note trabecular arrangement and intensive vacuolation within cytoplasm of tumour cells. Male mouse, 82 weeks old. H & E. ×140.

FIG. 4.—Hepatoma, glandular type. Note formation of acini and deposit of fibrous tissue. Male mouse, 95 weeks old. H. & E. ×140.

FIG. 5.—Hepatoma, trabecular type. Note severe pleomorphism and giant cell (centre). Female mouse, 87 weeks old. H. & E. ×140.

latent periods of the hepatic tumours are given in Table II. Group 1 and 2 males and females developed a significant incidence of hepatomas. Although Group 1 females had a higher percentage of hepatomas than did males (87% *vs* 83·3%) and Group 2 males had higher percentages of these neoplasms than females (68% *vs* 53·6%), the differences between groups and between the sexes were not significant. Males in Groups 3 and 4 developed only 3 hepatomas (2, Group 3; 1, Group 4) but no control animals had these tumours.

Histologically, liver tumours were either well-differentiated and solid with a structure similar to that of normal liver (Fig. 2), less differentiated trabecular (Fig. 3) or (occasionally) glandular (Fig. 4), in which case the liver structure was completely obliterated. The tumour cells in the well-differentiated neoplasms were arranged in 1–2 cell-thick plates similar to those of the normal liver cords. The intervening sinusoids were narrowed or obliterated because of expanding large neoplastic cells. However, in some areas, particularly in the central portions of large nodules, tumour cells were arranged in small clusters and separated by wide sinusoidal spaces often containing large quantities of blood. The surrounding parenchyma was commonly compressed (Fig. 2) owing to the expansive growth of these tumours. The transition from tumour to the surrounding normal hepatic tissue was sometimes poorly defined. The less-differentiated, trabecular-type tumours consisted of irregular cords several cells thick, and were divided by wide (sometimes narrow) vascular spaces. Occasionally the glandular type of tumour formed acini (Fig. 4) in association with extensive fibrosis.

Cytological irregularities included conspicuous hyalin and refractile eosinophilic inclusion bodies, intranuclear eosinophilic inclusion bodies, vacuolization of cytoplasm (Fig. 3), frequent basophilia, sometimes extensive pleomorphism and anisokaryosis (Fig. 5). These cytological features were more apparent in the less-differentiated neoplasms. No extra-hepatic metastases were noted.

*Other tumours.*—There was no significant difference in incidence of tumours other than hepatocellular neoplasms, between treated and control mice. Although there was an increased percentage of lung tumours among Group 3 males (28·0% *vs* 16·3% in controls) and of malignant lymphomas among Group 3 females (35·7% *vs* 27·6%), the increase in both tumour types was not significant.

*Rats*

Table I gives treatment and survival rates for test and control rats. In the first 3 weeks of the experiment, the rats tolerated the drug well, but thereafter displayed such signs of toxic effect as somnolence, sometimes slight icterus, and abdominal distention. Toxic effects, although more prominent in male rats, were less intense than in mice, since no animals died within the first 10 weeks of the experiment. Livers showed traces of golden-brown pigment (probably protoporphyrin) in the Kupffer cells, and a virtual absence of such material in liver parenchymal cells.

*Thyroid tumours.*—The frequency and percentage of tumours are given in Table III. The incidence of thyroid tumours in Group 1 males (53·3%) and females (23·3%) was highly significant ($P \leqslant 0{\cdot}001$) when compared with the spontaneous incidence in controls (1% for males and 2% for females). Group 2 rats developed fewer thyroid tumours (36·7%) and females 26·7%, which was still significant for both sexes ($P \leqslant 0{\cdot}001$). The percentage of thyroid tumours in Group 3 was considerably lower [13·3% in males, statistically significant ($P \leqslant 0{\cdot}02$), and 6·7% in females]. The ratios of thyroid tumours at the 3 consecutive dose levels indicated a classic dose-response relationship. Grossly, the thyroid tumours varied in size and shape. Large nodules associated with enlargement of the entire lobe were rare. Macroscopically, nodules ranged from 3–10 mm in diameter and appeared well-

† Peripheral nervous system.

Fig. 6.—Adenoma, follicular type, of thyroid gland. Note hyperplastic epithelium, abundance of colloid in macrofollicular and occasional psammoma bodies. H. & E. ×80.

Fig. 7.—Adenoma, micro- and macrofollicular pattern of architecture. Note dense areas of neoplastic tissue and compression of normal follicles at right. H. & E. ×80.

Fig. 8.—Carcinoma of thyroid, papillary type; neoplastic tissue exhibiting both follicular and papillary structural arrangement. Note short papillary structure into cystic cavitation. H. & E. ×160.

Fig. 9.—Carcinoma of thyroid, follicular type. Note few microfollicular structures and massive invasion of neoplastic tissue in fibrous capsules (arrow). H. & E. ×100.

circumscribed. Histologically, the thyroid tumours were predominantly adenomas, consisting of follicles, which were either of macrofollicular (Fig. 6) or macro- and microfollicular types (Fig. 7) and/or of papillary-cystic structures. Since many thyroid adenomas had features of both types of neoplasm, they could be termed mixed thyroid adenomas. The colloid material was usually abundant in macrofollicles and cysts, but appeared scarce or absent in the microfollicles. The follicular epithelium of thyroid tumours was cuboidal and uniform in shape, but more flattened in macrofollicles and cysts. Most thyroid-tumour-bearing animals had cystic cavitations containing various amounts of colloid material within the lesions (in over 70% of all cases). In numerous instances, solitary cysts with colloidal material were found in the thyroid glands of non-tumour-bearing animals. It would appear that the drug exerted some goitrogenic effect on the thyroid.

Thyroid carcinomas with varying degrees of anaplasia were of either papillary or follicular types. Some of the carcinomas had distinct papillary projections into the cystic spaces (Fig. 8) and contained sparse amounts of colloidal material. The follicular type of carcinoma was characterized by small follicles lined by cells having dense nuclei with coarse chromatin. The microfollicles were usually devoid of colloidal material. Carcinomas had thick capsules invaded by neoplastic cells (Fig. 9). No distant metastases of thyroid tumours were found. There were 4 carcinomas (in 3 males and 1 female) in Group 1 and 3 (2 males and 1 female) in Group 3. Three tumours in controls were benign thyroid adenomas. One medullary carcinoma of the thyroid deriving from C cells was encountered in a thyroid-adenoma-bearing animal.

*Other tumours*.—Table III lists other tumours in rats. Group 2 animals had an increased incidence of pituitary gland tumours, predominantly in males, mammary tumours in females, and interstitial-cell tumours of the testes. A squamous-cell tumour of the skin, and tumours of the peripheral nervous system were present in Group 1. Pituitary tumours were mainly adenomas but there were a few carcinomas. Many of these tumours (over 50%) were associated with thyroid-tumour-bearing animals. Over 60% of the females with mammary tumours had pituitary adenomas, but the latter type occurred at an even higher rate (70%) in controls. The prevailing types of mammary tumours were fibroadenomas, adeno-fibromas and adenomas. Several mammary tumours were adenocarcinomas, which appeared in both control (5) and test (7) groups. The parathyroid tumours in both control and test animals were benign adenomas. The incidence of these tumours in Group 3 does not appear significantly increased when compared with the parathyroid tumours occurring in controls. The skin tumours in Group 1 males were 3 squamous-cell papillomas, one tricho-epithelioma and 3 squamous-cell carcinomas, while controls had 6 squamous cell papillomas and 1 squamous-cell carcinoma.

A number of other tumour types in test and control rats (Table III) were representative of lesions commonly found in untreated Eppley rats.

*Hamsters*

The treatment and survival rates of experimental and control hamsters are given in Table III. Compared to mice and rats, the hamsters tolerated griseofulvin well, and showed no excessive toxic effects.

The incidence of tumours, their distribution and origin showed no significant differences between test and control hamsters. The tumour types by group (and latency in weeks) are as follows: Group 1, females—1 fibrosarcoma of subcutis (52); males—3 adenomas of adrenal cortex (100, 116, 124), 1 adenocarcinoma of thyroid (102), 1 sarcoma of subcutis (106), 1 malignant lymphoma, histiocytic type (119); Group 2, females—1 granulosa-cell tumour of ovary (94); males—2 adenomas of adrenal cortex (117, 120), 1 adenoma of kidney (99); Group 3, females—1 cholangiocarcinoma of liver (87), 1 granulosa-theca-cell tumour of ovary (83), 1 adenocarcinoma of uterus (101), 1 squamous cell carcinoma of skin (87); males—malignant lymphoma, histiocytic type (118); Group 4, females—2 papillomas of forestomach (75, 88), 1 haemangioma of ovary (88), 1 squamous-cell carcinoma of uterus (99), 1 haemangioma of liver (96), 1 haemangioma of spleen (96), 1 adenocarcinoma of uterus (99), 1 adenocarcinoma of adrenal cortex (99), 1 carcinoma of thyroid (78), 1 myxosarcoma, retroperitoneal (92); males—2 pheochromocytomas of adrenal gland (116, 116), 2 malignant lymphomas, histiocytic type (87, 100), 2 fibrosarcomas of subcutis (76, 84), 1 papilloma of forestomach (114), 1 adenoma of adrenal cortex (105), 1 adenoma of gall bladder (84).

### DISCUSSION

Dietary exposure to griseofulvin produced a significant incidence of hepatocellular tumours in mice, thyroid tumours in rats, but no carcinogenic activity in hamsters.

The total number of hepatomas was particularly significant in mice fed at the 2 highest dose levels, while the rates of hepatocellular tumours decreased proportionately at lower concentrations, indicating a dose response for the incidence of neoplasms in experimental groups. The hepatocarcinogenic effect of griseofulvin and the manifestation of a dose response were reported by Hurst and Paget (1963) who used only 2 dose levels. Subsequently, De Matteis *et al.* (1966) found a pronounced sex difference in the hepatocellular tumour incidence of mice exposed to griseofulvin, in that males not only showed a greater incidence and multiplicity of these tumours, but also a higher degree of porphyria and more pronounced histological changes in liver. Similarly, hepatic-cell tumours have occurred in male mice exposed to milligram quantities of griseofulvin at birth and infancy (Epstein *et al.* 1967). The exclusive hepatocarcinogenic effects of griseofulvin in mice in the present experiment are consistent with results in different strains of adult mice (DeMatteis *et al.*, 1966; Hurst and Paget, 1963), and with those of Epstein *et al.* (1967) in newborn or infant mice. At variance with previous reports (DeMatteis *et al.*, 1966; Epstein *et al.*, 1967) is the production in the present experiment of hepatocellular tumours in both female and male mice.

Little is known of the mechanisms by which griseofulvin is hepatocarcinogenic in mice. Hurst and Paget (1963) suggested that massive deposits of protoporphyrin in finer ramifications of the biliary tract caused biliary cirrhosis, an alteration that could result in neoplasia. However, the strong hepatocarcinogenic effect of the drug in infant mice dosed briefly parenterally (Epstein *et al.*, 1967) indicates an involvement of griseofulvin with cellular constituents (nucleic acids or proteins).

Recently it was suggested that hepatoma induction in mice cannot be held as a valid demonstration of carcinogenicity (Grasso and Crampton, 1972). Tomatis *et al.* (1973), however, indicated that induction of liver-cell tumours in mice should be considered as valid a demonstration of carcinogenicity as tumour induction in rats and hamsters.

The finding of thyroid tumours in rats is especially interesting, because in previous studies (Paget and Walpole, 1960; Paget and Alcock, 1960) with this antimitotic drug, no such tumours were reported. These neoplasms have been induced by irradiation (Lindsay *et al.*, 1961; Nichols *et al.*, 1965; Lindsay *et al.*, 1968; Money *et al.*, 1965), administration of goitrogens (Money *et al.*, 1965), and by maintaining animals on an iodine-deficient diet. The mechanism of genesis for these tumours is unknown, but in our study over 70% of thyroid-tumour-bearing animals had cystic dilatations containing colloid material, which suggests a possible goitrogenic effect. It is, of course, a matter of speculation whether such an effect may be associated with tumour induction by griseofulvin.

The carcinogenic activity of griseofulvin, as corroborated in this experiment, was formerly limited to mouse liver (DeMatteis *et al.*, 1966; Lochhead *et al.*, 1967). While previous reports in rats (Klein and Beall, 1972; Paget and Alcock, 1960) have yielded little information about carcinogenicity (perhaps owing to their brevity), our study records positive results in this species. However, further experimental data in rats on the mechanism of induction of thyroid tumours and other neoplasms, including the use of other biological systems for carcinogenicity assay, are required, as are epidemiological studies in man.

This work was supported by Public Health Service contract NO1 CP33278 from the Division of Cancer Cause and Prevention, National Cancer Institute. We thank Sue Ginder and Mara Payich for excellent technical assistance, Mardelle Susman for editorial help and Andrew Washington and Walter Williams for preparing the illustrations.

### REFERENCES

ANDERSON, D. W. (1965) Griseofulvin: biology and clinical usefulness. *Ann. Allergy*, **23**, 103.

ANONYMOUS (1966) Effects of griseofulvin in mycotic infections of the scalp. *W.H.O. Chron.*, **28**, 310.

BALLO, J. M. & CUTTING, R. T. (1970) A field trial of griseofulvin in the prophylaxis of dermatophytoses. In *Annual Progress Report*, Vol. 2, Washington: Walter Reed Army Inst. Res., p. 1109.

BARICH, L. L. & BARICH, D. (1963) Conversion of antitumor into tumor promoting effect with prolonged intake of oral griseofulvin in methylcholanthrene painted mice. *Clin. Med.*, **70**, 590.

BARICH, L. L., NAKAI, T., SCHWARZ, J. & BARICH, D. J. (1960) Tumor-promoting effect of excessively large doses of oral griseofulvin in tumors induced in mice by methylcholanthrene. *Nature*, **187**, 335.

BARICH, L. L., SCHWARZ, J. & BARICH, D. (1962) Oral griseofulvin: a cocarcinogenic agent to methylcholanthrene-induced cutaneous tumors. *Cancer Res.*, **22**, 53.

BARICH, L. L., SCHWARZ, J., BARICH, D. J. & HOROWITZ, M. G. (1961) Toxic liver damage in mice after prolonged intake of elevated doses of griseofulvin. *Antibot. Chemother.*, **11**, 566.

BEARE, J. M., GENTLES, J. C. & MACKENZIE, D. W. R. (1968) Griseofulvin. In *Textbook of Dermatology.* Eds A. Rook, D. S. Wilkinson and F. J. C. Ebling. Oxford: Blackwell, p. 904.

BLANK, H., SMITH, J. G., ROTH, F. J., JR, & KANENSON, W. (1959) Griseofulvin for the systemic treatment of dermatomycoses. *J. Am. Med. Assoc.*, **171**, 2168.

DEMATTEIS, F. (1963) Disturbance of porphyrin metabolism caused by griseofulvin in mice. *Br. J. Dermatol.*, **75**, 91.

DEMATTEIS, F., DONNELLY, A. J. & RUNGE, W. J. (1966) The effect of prolonged administration of griseofulvin in mice with reference to sex differences. *Cancer Res.*, **2**, 271.

EALES, L. (1963) The effect of griseofulvin in acute porphyria. *S. Afr. J. Lab. Clin. Med.*, **9**, 304.

EPSTEIN, S. S., ANDREA, J., JOSHI, S. & MANTEL, N. (1967) Hepatocarcinogenicity of griseofulvin following parenteral administration to infant mice. *Cancer Res.*, **27**, 1900.

EPSTEIN, W. L. & LARSON, M. A. (1961) Griseofulvin potentiation of colchicine toxicity. *J. Invest. Dermatol.*, **36**, 5.

GRASSO, P. & CRAMPTON, R. F. (1972) The value of the mouse in carcinogenicity testing. *Food Cosmet. Toxicol.*, **10**, 418.

GRIN, E. L. (1965) A controlled trial of home *versus* hospital treatment of tinea capitis with griseofulvin. *Bull. W.H.O.*, **33**, 193.

HURST, W. E. & PAGET, G. E. (1963) Protoporphyrin, cirrhosis and hepatoma in the livers of mice given griseofulvin. *Br. J. Dermatol.*, **75**, 105.

KLEIN, M. F. & BEALL, J. R. (1972) Griseofulvin: a teratogenic study. *Science*, **175**, 1483.

LINDSAY, S., NICHOLS, C. W., JR & CHAIKOFF, I. L. (1968) Carcinogenic effect of irradiation. *Arch. Pathol.*, **85**, 487.

LINDSAY, S., SHELINE, G. E., POTTER, G. D. & CHAIKOFF, I. L. (1961) Induction of neoplasms in the thyroid gland of the rat by X-irradiation of the gland. *Cancer Res.*, **21**, 9.

LOCHHEAD, A. C., DAGG, J. H. & GOLDBERG, A. (1967) Experimental griseofulvin porphyria in adult and foetal mice. *Br. J. Dermatol.*, **79**, 96.

MONEY, W. L., TYPOND, P. & RAWSON, R. W. (1965) The growth and function of thiouracil-induced thyroid tumors transplanted into non-inbred rats thymectomized at birth. *Cancer Res.*, **25**, 423.

NICHOLS, C. W. JR, LINDSAY, S., SHELINE, G. E. & CHAIKOFF, I. L. (1965) Induction of neoplasms in rat thyroid glands by X-irradiation of a single lobe. *Arch. Path.*, **80**, 177.

OXFORD, A. E., RAISTRICK, H. & SIMONART, P. (1939) Studies of the biochemistry of microorganisms. LX. Griseofulvin, $C_{17}H_{17}O_6Cl$, metabolic products of penicillium griseofulvin. *Biochem. J.*, **33**, 240.

PAGET, G. E. & ALCOCK, S. J. (1960) Griseofulvin and colchicine: lack of carcinogenic action. *Nature* **188**, 867.

PAGET, G. E. & WALPOLE, A. L. (1958) Some cytological effects of griseofulvin. *Nature*, **182**, 1320.

PAGET, G. E. & WALPOLE, A. L. (1960) The experimental toxicology of griseofulvin. *Arch. Dermatol.*, **81**, 152.

REDEKER, A. G., STERLING, R. E. & BRONOW, R. S. (1964) Effect of griseofulvin in acute intermittent prophyria. *J. Am. Med. Assoc.*, **188**, 466.

RIMINGTON, C., MORGAN, P. N., NICHOLLS, K., EVERALL, J. D. & DAVIES, R. R. (1963) Griseofulvin administration and porphyrin metabolism. A survey. *Lancet*, ii, 318.

Scott, F. W., Delahunta, A., Schultz, R. D., Bistner, S. I. & Riis, R. C. (1975) Teratogenesis in cats associated with griseofulvin therapy. *Teratology*, **11**, 79.

Tomatis, L., Partensky, C. & Montesano, R. (1973) The predictive value of mouse liver tumor induction in carcinogenicity testing—a literature survey. *Int. J. Cancer*, **12**, 1.